# Magnetic Resonance Phase Alterations in Multiple Sclerosis Patients with Short and Long Disease Duration

**DOI:** 10.1371/journal.pone.0128386

**Published:** 2015-07-17

**Authors:** Ivan Bozin, Yulin Ge, Joseph Kuchling, Petr Dusek, Sanjeev Chawla, Lutz Harms, Klemens Ruprecht, Thoralf Niendorf, Friedemann Paul, Ilya Kister, Tim Sinnecker, Jens Wuerfel

**Affiliations:** 1 NeuroCure Clinical Research Center, Charité –Universitaetsmedizin Berlin, Berlin, Germany; 2 Department of Radiology, NYU School of Medicine, New York, New York, United States of America; 3 Institute of Neuroradiology, Universitaetsmedizin Goettingen, Goettingen, Germany; 4 Department of Neurology and Center of Clinical Neuroscience, Charles University in Prague, 1st Faculty of Medicine and General University Hospital in Prague, Prague, Czech Republic; 5 Clinical and Experimental Multiple Sclerosis Research Center, Charité Universitaetsmedizin Berlin, Berlin, Germany; 6 Department of Neurology, Charité—Universitaetsmedizin Berlin, Berlin, Germany; 7 Berlin Ultrahigh Field Facility, Max Delbrueck Center for Molecular Medicine, Berlin, Germany; 8 Experimental and Clinical Research Center, Charité—Universitaetsmedizin Berlin and Max Delbrueck Center for Molecular Medicine, Berlin, Germany; 9 Multiple Sclerosis Care Center, Department of Neurology, NYU School of Medicine, New York, New York, United States of America; 10 Department of Neurology, Asklepios Fachklinikum Teupitz, Teupitz, Germany; Cornell University, UNITED STATES

## Abstract

**Objective:**

The analysis of the MR phase provides additional information on the tissue microstructure. In multiple sclerosis (MS) lesions phase alterations may reflect different stages of inflammatory activity. Here we investigated lesion morphology in MS patients with short and long disease duration on T2* weighted, phase, magnitude and susceptibility weighted imaging (SWI) at 7 Tesla (T).

**Methods:**

17 MS or clinically isolated syndrome patients with short (<60 months) and 11 with long (>60 months) disease duration underwent 7T MRI. Lesions were subsequently analyzed side-by-side with regard to morphology and visibility on T2* weighted, SWI, magnitude and SWI-filtered phase images.

**Results:**

126 of 192 T2* weighted lesions (65.6%) were characterized by a phase alteration pattern, and hence could be differentiated on phase images. In detail, a significantly reduced proportion of lesions showing phase alterations was detectable in patients with longer disease duration (mean±SD 51±37%, range 0–100%) compared to patients with short disease duration (mean±SD 90±19.5%, range 50–100%, p = 0.003).

**Conclusion:**

This cross-sectional study identified different patterns of phase changes in lesions of MS patients with short and long standing disease. Longitudinal studies are warranted to prove that MR phase imaging is useful in determining the activity and the developmental stage of individual MS plaques.

## Introduction

Multiple Sclerosis (MS) is an inflammatory, demyelinating and neurodegenerative central nervous system disease [1]. Magnetic resonance imaging (MRI) improved early MS diagnosis by demonstrating spatiotemporal lesion dissemination. However, conventional MR parameters are not specific for MS, and correlate moderately with clinical disability—a phenomenon termed “clinico-radiological paradox” [2]. Today, ultrahigh field MRI at 7 Tesla (T) visualizes focal MS lesions in great detail [3–5]. Gaining from increased signal to noise ratio and enhanced susceptibility effects, MS lesions on 7T T2* weighted (T2*w) images frequently display a small central vein. A proportion of lesions also exhibits a hypointense rim. Both characteristics can be used to distinguish MS from other white-matter pathologies such as neuromyelitis optica [5], Susac syndrome [6], and white matter lesions of presumably vascular origin [7,8], which consequently improves the diagnosis of MS [9].

Early reports [9–11] suggested that the T2* hypointense rim surrounding MS plaques is caused by microglia and macrophages containing iron—a paramagnetic metal involved in (repair-) processes that are abnormal within the MS brain [12]. However, the underlying pathomechanism causing susceptibility related signal loss at the edges of such lesions in T2*w MRI is still not fully understood. Recently, rim-like phase abnormalities in MS lesions were correlated with increased inflammatory activity of the evolving MS plaque [13].

Susceptibility induced magnetic resonance (MR) phase alterations may contain additional information on tissue microstructure [14], exceeding alterations in MR signal magnitude. However, the MR phase of the white matter is not only determined by the iron content and the degree of myelination, but also depends on the structural parenchymal integrity i.e. of axons and myelin bundles [14]. According to this hypothesis, MR phase may significantly shift during MS lesion formation.

Inspired by preliminary data on phase abnormalities in MS we performed 7T MRI with high spatial resolution to analyze lesion morphology in a cross-sectional study. For this purpose susceptibility weighted imaging (SWI) yielding high spatial resolution SWI-filtered phase images, and T2*w MRI were applied along with anatomical T1 weighted (T1w) and fluid attenuated inversion recovery (FLAIR) data. Furthermore, we compared measures of phase changes in MS patients with early versus long standing disease.

## Materials and Methods

### Subjects

We enrolled 28 MS patients (11 female), including four patients with clinically isolated syndrome (CIS), 22 patients with relapsing remitting disease course (RRMS), and two patients with primary progressive disease course fulfilling the current panel criteria [1]. Other radiographic features of some relapsing remitting and primary progressive MS cases included here had been reported previously [2,15–17]. 7T MR phase images of these patients were not published elsewhere. Disability was assessed using the Expanded Disability Status Scale (EDSS) [18]. Patients with CIS and MS with disease onset within less than 5 years were defined as “MS with short duration”, others as “MS with long duration”. Further details on the patient cohort are presented in Table 1. The study was approved by the local ethics committee (Ethics Commission of Charité—Universitätsmedizin Berlin—EA 1/054/09). Written informed consent was obtained from all subjects prior to the study.

**Table 1:** Cohort description.

	all patients	MS with short duration	MS with long duration
**n**	28	17	11
**Gender [n = female]** ^**§**^	11	8	3
**Age [years] mean±SD, range** ^**$**^	34.8±8.0, 19–55	30.7±6.1, 19–43	41±6.6, 33–55
**Time since first symptoms [months] mean±SD, range**	69±69,2–201	20±18,2–58	144±47,62–201
**EDSS median, range**	1.5, 0–7.0	1.5, 0–3	1.5, 1–7
T2*w lesion count [n],mean±SD, range	7.11±7.28,0–26	5.12±6.81,0–26	9.55±7.49,1–21
**T1 hypointense lesion count [n], mean±SD, range**	7.11±7.28,0–26	5.12±6.81,0–26	9.55±7.49,1–21
**SWI lesion count [n], mean±SD, range**	6.04±7.37,0–26	4.59±6.59,0–26	8.5±8.32,1–21
**Magnitude lesion count [n], mean±SD, range**	5.93±7.31,0–26	4.29±6.41,0–26	8.7±8.22,1–21
**Phase lesion count [n], mean±SD, range**	4.67±5.46,0–20	4.17±4.91,0–15	5.5±6.47,0–20
**Phase visibility [%], mean±SD, range** ^**#**^	74.3±33,0–100	90±19.51,50–100	51±37,0–100

Patient-wise analysis. Key: MS with short duration: MS patients with less than 5 years disease duration; MS with long duration: MS patients with more than 5 years disease duration; SD: standard deviation

§ p = 0.295, Pearson’s Chi-squared test to assess gender differences between MS with short and long duration.

$ p<0.001, Kruskal-Wallis test to assess age differences between MS with short and long duration.

# p = 0.003, Mann-Whitney U test to assess group differences between MS with short and long duration.

### MRI data acquisition

Ultrahigh field MR images were acquired using a 7T whole body MR scanner (Magnetom, Siemens, Erlangen, Germany), applying a 24-channel receive head coil (Nova Medical, Wilmington, MA, USA) equipped with a birdcage volume coil used for transmission. The imaging protocol included 2D T2*w fast low angle shot (FLASH, TE = 25.0ms; TR = 1820ms; spatial resolution = (0.5 x 0.5 x 2.0) mm^3^), 3D T1w magnetisation prepared rapid gradient echo (MPRAGE, TE = 2.98ms; TR = 2300ms; inversion time = 900ms; flip angle = 5°; spatial resolution = (1.0 × 1.0 × 1.0) mm^3^), two dimensional fluid attenuated inversion recovery (FLAIR, TE = 90ms; TR = 16000ms; TI = 2925.5ms; spatial resolution = (1.0 × 1.0 × 3.0) mm^3^), and 3D gradient echo flow-compensated susceptibility weighted imaging (SWI, TE = 14ms; TR = 25ms; flip angle = 12; spatial resolution = (0.5 x 0.5x 1.0 mm)^3^) yielding magnitude, SWI-filtered phase and reconstructed SWI images.[19] SWI, phase and magnitude images of one a MS patient with long disease duration needed to be excluded due to low image quality.

### Image analysis

**Fig 1:**
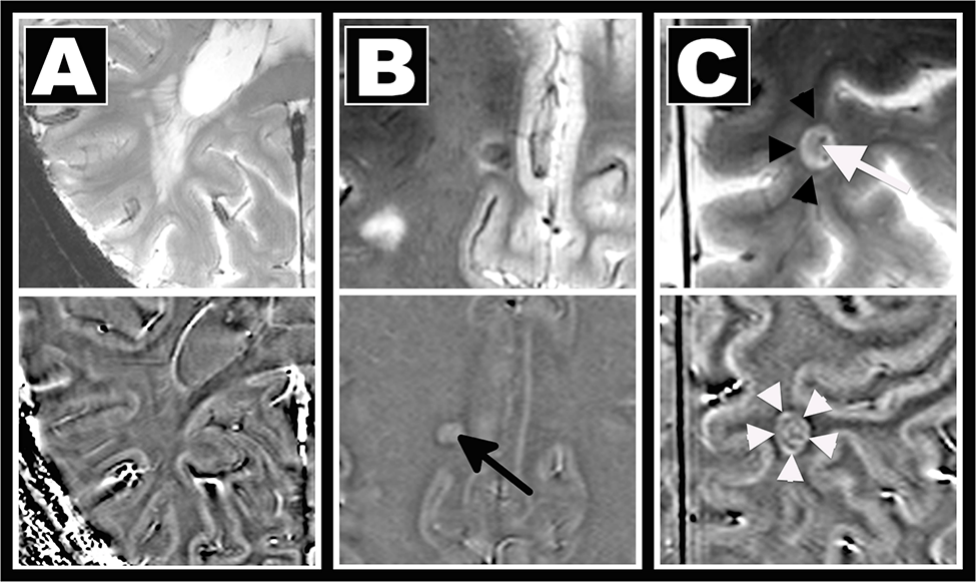
Methods. MS lesion morphology was assessed on T2*w (top) and phase (bottom) images using a visual analysis. Lesion visibility, the existence of a central vein, and signal alterations indicative for iron deposition were rated. A: confluent T2*w hyperintense lesion not visible on phase images. B: central T2*w hypointensity and positive phase shift indicative of iron deposits (arrow). C: Iron deposits in a perivascular (white arrow) lesion causing a T2*w hypointense rim (black arrowheads) and ring-like paramagnetic (“bright”) phase alterations (white arrowheads). Please note that the dark envelop around the “bright” ring in the phase image likely represents an artifact caused by the dipolar field patterns.

MRI images were analyzed using OsiriX (OsiriX Foundation, Genève, Switzerland, version 3.8.1). Each lesion was first marked by two experienced investigators (IB and TS) in consensus reading on high spatial resolution T2*w FLASH images that depicts neuroinflammatory lesions with great anatomical details. For this purpose a lesion was defined as a hyperintensitiy of at least 3 voxel. T2*w hypointensities were also assessed and subsequently confirmed on FLAIR images. The latter were also used to exclude enlarged perivascular spaces. [20] T2*w hyperintense lesions without corresponding FLAIR signal alteration were only marked as MS lesions in the case of 7T MR lesion characteristics typical for MS. [4–9]Next, lesions with a diameter of at least 5mm were characterized in more detail side-by-side on each sequence for each imaging technique. For this purpose T2*w lesions were marked on T1w, SWI, magnitude, SWI-filtered phase and FLAIR images. Phase alterations were analyzed on SWI-filtered phase images only. In detail, we assessed i) the visibility of a given T2*w hyperintense lesion on each sequence, ii) the occurrence of a strong signal hypointensity on T2*w/SWI images with corresponding positive (paramagnetic) phase shift as marker of iron deposits within the center of the lesion or the rim, and iii) the presence of a small central vein (Fig 1).

Please note that our scanner uses the left-handed system where paramagnetic substances like iron cause positive phase shift and thus appear bright on phase images. This perspective is kept throughout the text. White matter lesions were differentiated into subcortical (distance to cortical grey matter ≤2 mm, including leukocortical lesions), periventricular (distance to ventricle ≤5 mm), and other white matter (distance to ventricle >5 mm and distance to cortical grey matter >2 mm) lesions. Given the complex mechanisms of phase contrast within the grey matter, this 7T MRI pilot study was focused on white matter alterations. Thus purely intra-cortical or subpial cortical lesions were not assessed.

### Statistical Analysis

All analyses were performed in IBM SPSS Statistics (version 20, IBM, Somers, NY, USA). P-values <0.05 were considered significant. Group differences between MS with short and long duration were assessed using non-parametric Mann-Whitney U test. Gender differences were analyzed using Pearson’s Chi-squared test, and age differences between MS with short and long duration were described using Kruskal-Wallis test. Dependencies between the variables examined were studied using non-parametric Spearman correlation. All tests should be understood as exploratory data analyses, such that no previous power calculation and adjustments for multiple testing were performed.

## Results

In total, 826 white matter lesions were detected in 28 patients on magnitude T2*w and subsequently confirmed on FLAIR images. Of these, 184 hyperintense and 8 hypointense plaques were larger than 5mm in diameter, and thus included in a detailed analysis. Two MS patients with short duration did not show any lesions larger than 5mm in diameter. As expected, the majority of these lesions were found in a periventricular localization (n = 96, 50%) and 34 lesions (18%) were classified as other white matter lesions. 62 lesions (32%) were located in the subcortical white matter. Of these, 24 lesions involved the cortical grey matter and were hence defined as leukocortical lesions. Purely intra-cortical lesions were–as stated above–not specifically assessed in this study.

### Lesion detection on 7T MR sequences

Each of the 192 T2*w lesions was subsequently marked on T1w, FLAIR, and SWI with the latter providing magnitude and phase images. All lesions were characterized by a prominent T1w hypointensity on the 3D T1w dataset, as described previously [2]. On FLAIR, 187 MS lesions (97%) were detectable, however, only a proportion of these, 160 lesions (83%), was simultaneously visible on SWI magnitude images. Fusing magnitude and phase to an SWI did not improve the detectability of these lesions (n = 163, 85%). Only a subgroup of all lesions was characterized by phase abnormalities and thus visualized on SWI-filtered phase images (n = 126, 66%). Further details are summarized in Table 1.

### Morphology of MS lesions on SWI-filtered phase images

It is conceivable that the detectability of lesions on phase images is related to the lesion size. However, we could not observe such correlations. Not all large MS plaques necessarily exhibited a strong phase shift, some of these lesions did not show any phase abnormalities at all (S1 Fig). Hence, lesions showing phase changes were not larger compared to lesions not detectable on phase images (p = 0.825, lesions with phase changes mean±SD lesion size 7.5±2mm, range 5.1–16.9 mm; lesions without phase alterations mean±SD lesion size 7.5±2mm, range 5.0–13.8mm).

**Fig 2:**
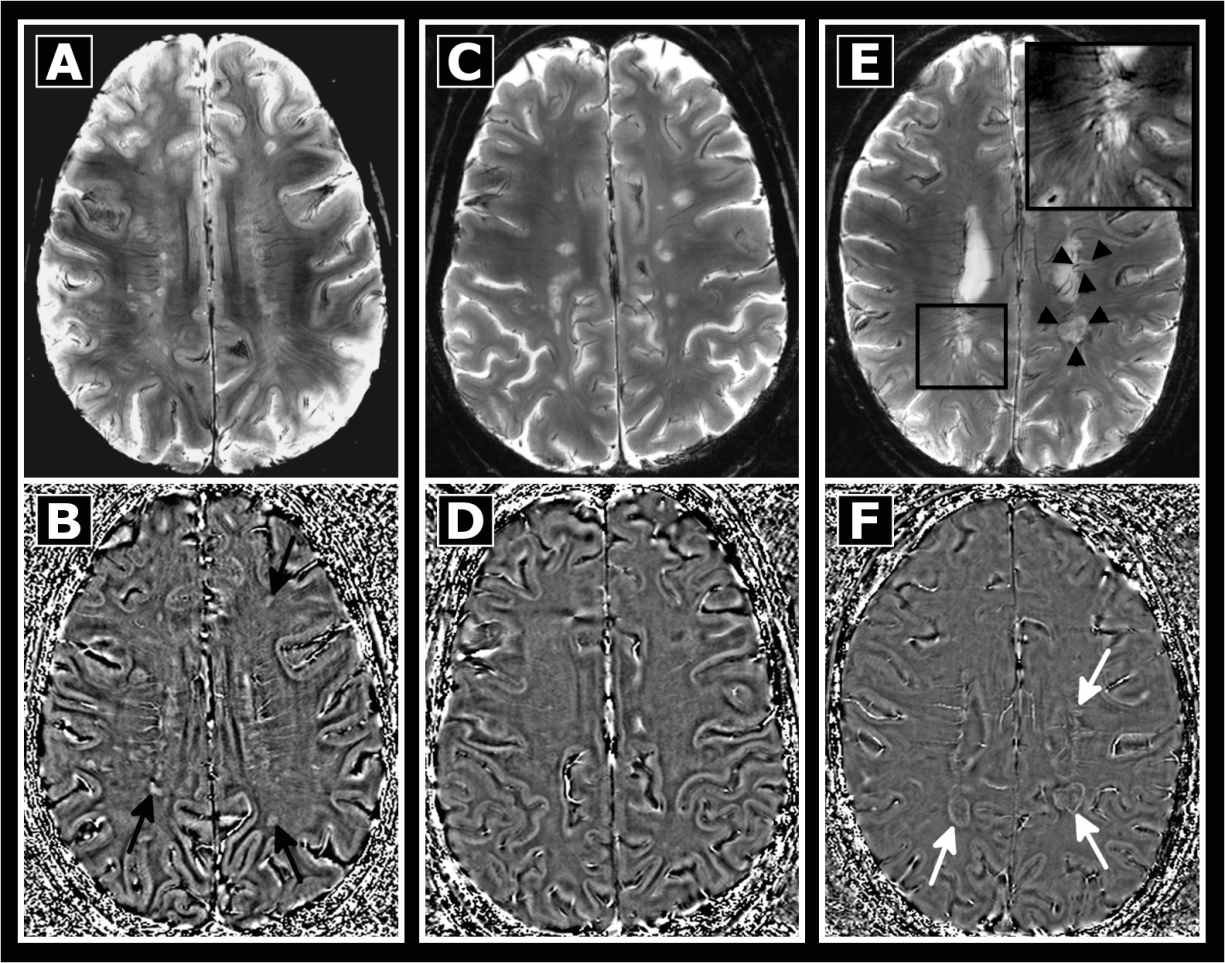
Comparison between early and long standing MS. T2*w (top) and phase images (bottom) of three patients are displayed. T2*w lesions (A) are detectable on phase images (B, black arrows) in the MS patient shown on the left (disease onset 9 months prior to MRI). Contrarily, no phase contrasting lesions were found in a patient with longer disease duration (C, D, MS onset 169 months prior to MRI). In addition, only a subgroup of patients with longer disease duration presented lesions with strong T2*w hypointense rims (E, black arrowheads, zoom) and corresponding rim-like phase abnormalities (F, white arrows).

**Fig 3:**
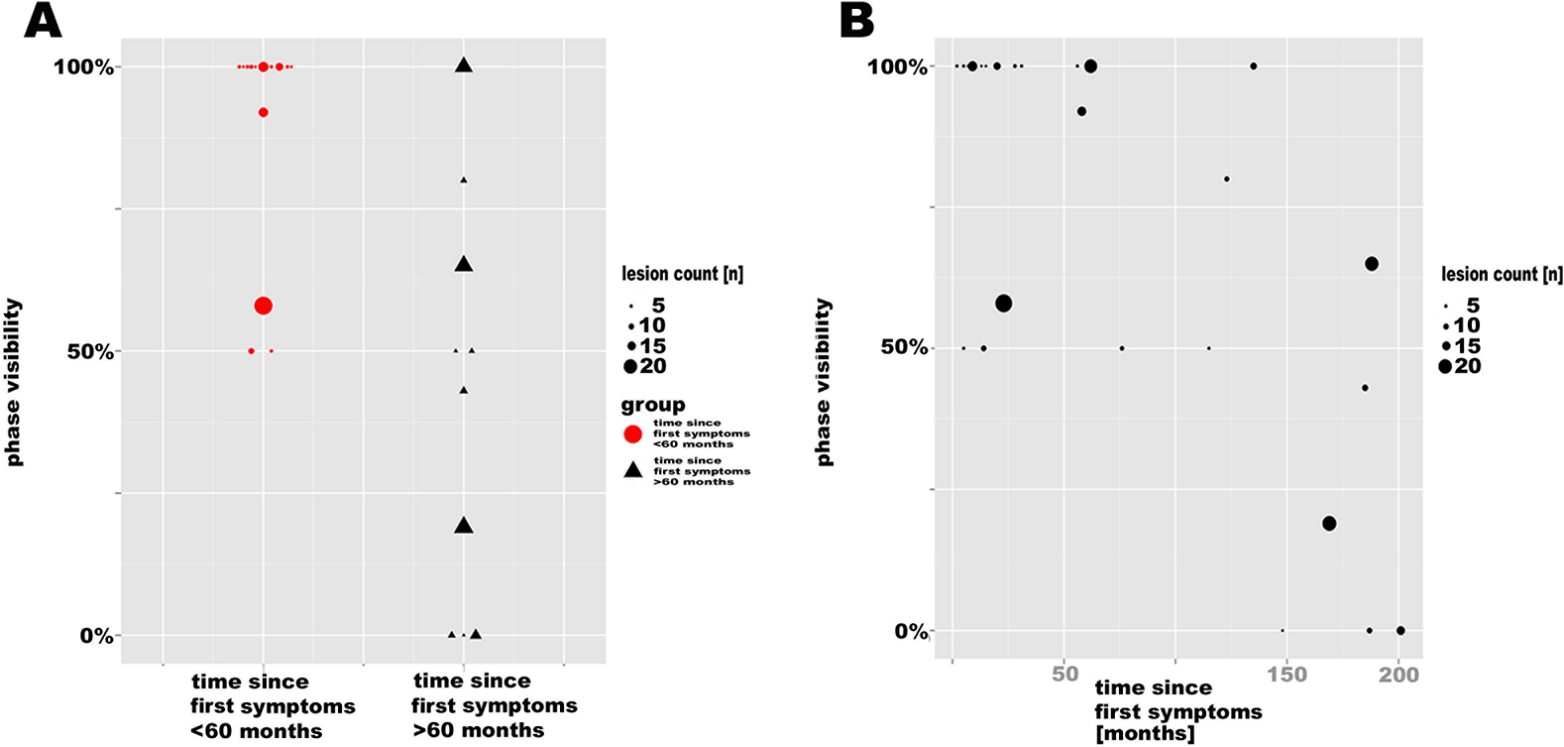
Scatter plot. A. The higher proportion of T2*w lesions visible on phase images in early compared to chronic disease stages is displayed. B. The scatter plot indicates an inverse correlation between phase visibility and disease duration.

**Fig 4:**
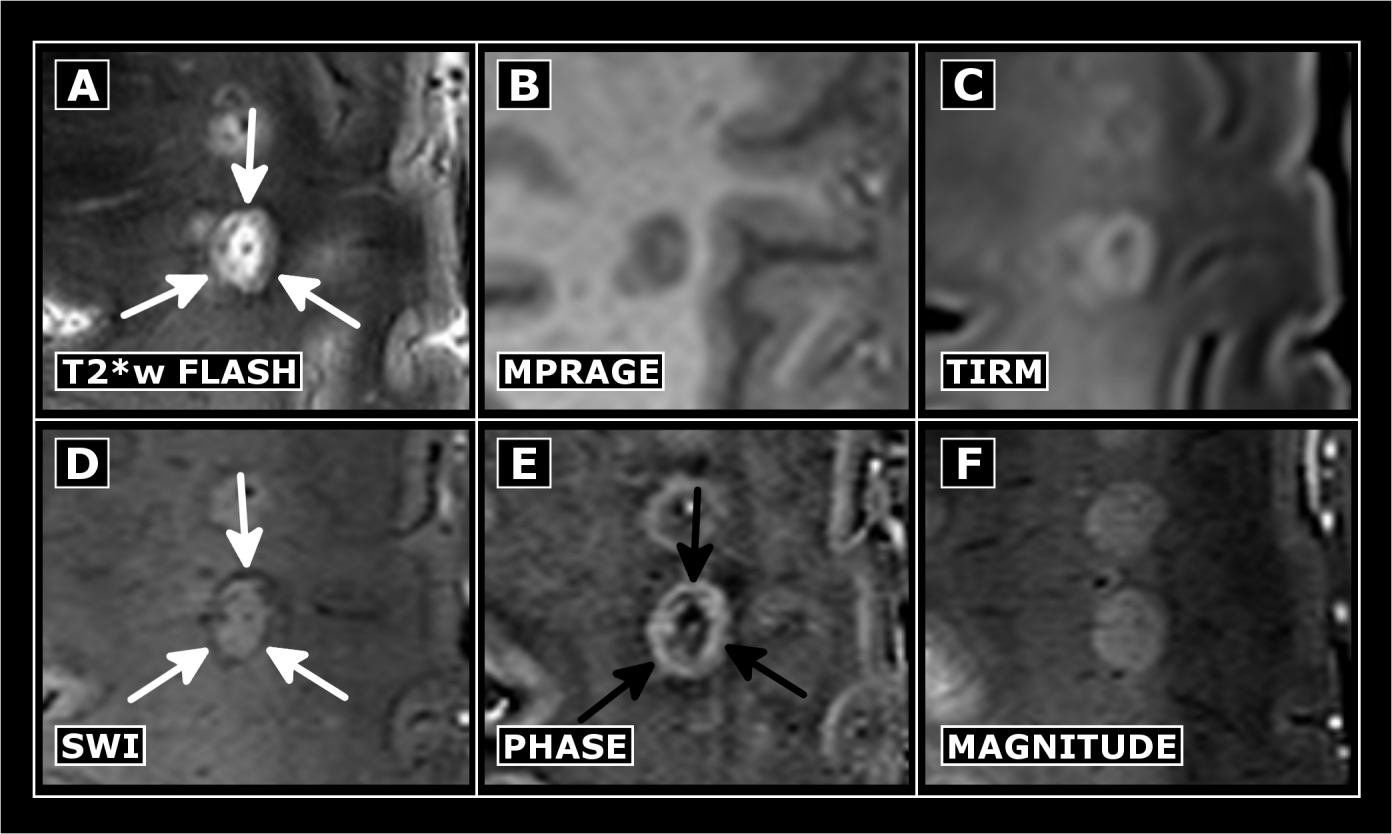
Exemplary chronic MS lesion. MS lesion in T2*w (A), T1w (B), FLAIR (C), SWI (D), phase (E) and magnitude (F) images. Prominent phase (black arrows) and corresponding T2*w/SWI hypointense rim are highlighted (white arrows).

Next, we examined whether the frequency of phase alterations in lesions correlated with disease duration. In a first visual analysis we found that lesions in long-standing MS patients were rarely characterized by phase alterations and thus only infrequently visualized on phase images (Fig 2). We further quantified phase visibility as ratio of lesions on T2*w and phase images in each patient. In MS patients with short disease duration, a higher proportion of lesions showed phase alterations (mean±SD phase visibility 90±20%, range 50–100%, Figs 2 and 3) than in patients with long duration (mean±SD 51±37%, range 0–100%, p = 0.003), where lesional phase changes were often related to a strong T2*w hypointense rim (30/192 lesions, median lesions per patient 1, range 0–6, Figs 2 and 4).

Furthermore, the visibility of lesions on phase images correlated inversely with disease duration, i.e. a lower proportion of lesions with phase alterations was associated with longer disease duration (r = -0.626, p = 0.001, Fig 3), and a trend towards higher EDSS scores in patients with a lower proportion of lesions with phase alterations (r = -0.357, p = 0.074). In addition, lesion visibility on phase images was inversely associated with age (r = -0.605, p = 0.001), but not with gender (p = 0.535).

### Iron deposits in MS lesions

**Fig 5:**
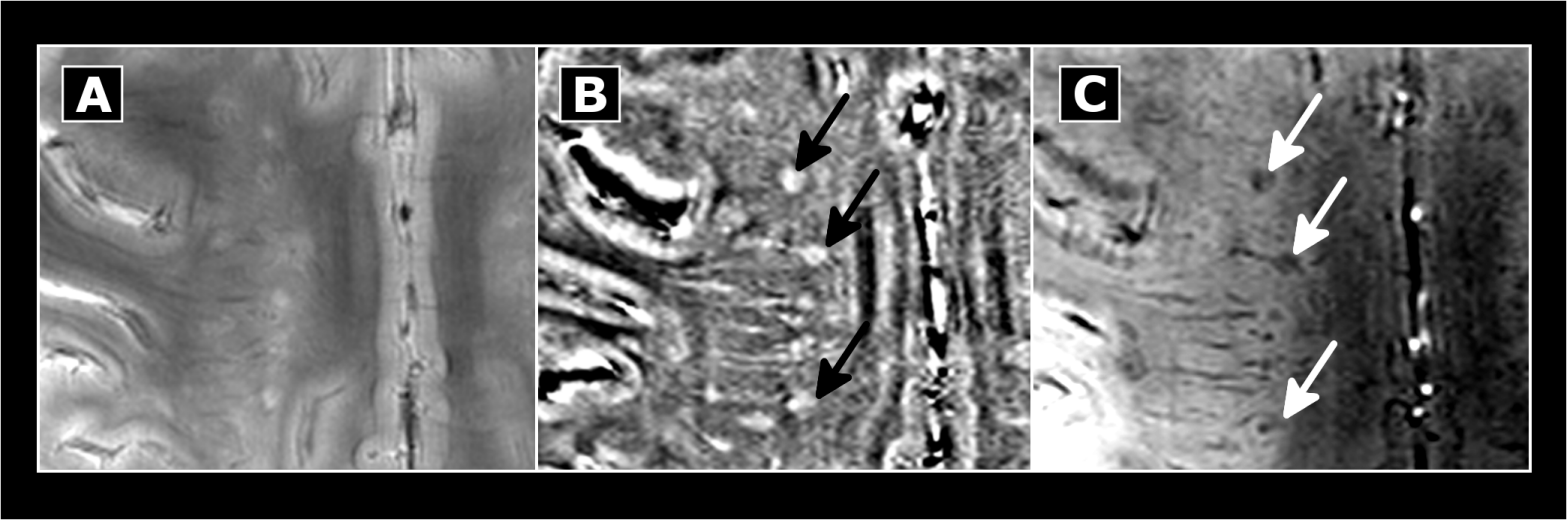
Phase abnormalities in MS lesions of patients with short disease duration. T2*w hyperintense (A) lesions of MS patients with short disease duration may be characterized by central phase shift (B, black arrows), and hypointense appearance on SWI (C, white arrows). Please note the higher sensitivity of SWI (C) compared to T2*w images (A) in detecting such alterations that might represent iron deposits.

Some lesions in patients with short disease duration were characterized by a positive (paramagnetic) phase shift and by a hypointense signal on SWI images—a feature reported to represent iron deposition within the center of MS lesions (Fig 5) [21]

In total, we detected phase abnormalities suggestive for iron deposits in 25 out of 192 lesions (10 of 25 patients; median number of lesions with presumable iron deposits 0, range 0–6). The SWI implementation was more sensitive in detecting alterations suggestive for iron within these lesions (n = 25) compared to the setup used for T2*w imaging (n = 8). Furthermore, central iron depositions were often detectable in lesions of MS patients with short duration (median number of lesions with iron deposits 1, range 0–6 detectable in 10/17 patients), but it was only infrequently seen in lesions in later disease stages (median number of lesions with iron deposits 0, range 0–3 detectable in 2/11 patients—one of them with only five years disease duration). This difference was, however, not statistically significant (p = 0.082).

### The central vein sign

The vast majority of lesions was characterized by a clearly visible central vein on T2*w images (n = 178, 93%). SWI and magnitude images provided excellent visibility of this characteristic feature of MS lesions (SWI n = 148, 91%; magnitude n = 140, 88%). Phase abnormalities suggestive for a central vein were, however, only detectable in 83 (66%) lesions. Due to sequence characteristics, veins could not be reliably analyzed on T1w and FLAIR images.

## Discussion

In this ultrahigh field MRI study we systematically analyzed phase abnormalities in lesions of MS patients with short and long disease duration. Only a subgroup of lesions was detectable on phase images acquired at 7T with a spatial resolution as good as 0.25 mm^3^. The proportion of phase alterations of lesions in early disease differed from that in later disease stages—in MS patients with long standing disease, white matter lesions were barely detectable in SWI-filtered phase images.

In general, tissue properties causing a phase shift do not necessarily correlate with changes in MR magnitude images, and vice versa. Phase abnormalities may provide additional information on the tissue microstructure. Factors determining phase changes are i.e. iron [22–24]^,^ myelin [24], cyto-architectural and axonal structures including neurofilament related anisotropy [14,25], soluble factors and proteins [13], or deoxyhemoglobin [26–28], all of which are involved in MS pathology.

Consequently, the interpretation of phase images remains hypothetical, given the fact that multiple variables contribute to phase changes within or at the edge of MS plaques.

Nevertheless, different phase alterations in MS patients with early and long disease duration as cross-sectionally demonstrated here are probably related to potential differences in the age of brain lesions between the two cohorts. Indeed, recent studies indicate that phase changes in MS lesions are time and/or disease-stage dependent [13,14,29]. In detail, computer simulations demonstrated how small alterations within the myelin cyto-architecture may cause substantial alterations of the local MR phase early during the process of demyelination [14]. A report on serial MR phase imaging in five MS patients confirmed these theoretical considerations, revealing phase abnormalities that preceded changes on magnitude images in three early MS lesions [29]. Furthermore, 95% of contrast enhancing MS lesions were reported to cause phase alterations [13]. These alterations during relapses were related to a distinct rim at the edge of the lesion. These thin phase rims are not closely associated with corresponding T2*w or prominent SWI hypointense signal alterations and reflect spreading inflammatory activity including higher concentrations of proteins at the lesion edges, rather than iron deposition [13].

In a different set of early disease stage lesions, phase alterations correlated with iron deposits within the center of the lesion [10,30–34]. Central iron deposition may result from blood brain barrier breakdown and subsequent perivascular hemoglobin leakage and degradation [10,32]. Therefore, focal intra-lesional iron deposits were suggested as in vivo marker in MS, grading the destructive capacity [35]. Another hypothesis refers to iron accumulation as a consequence of damaged and dying oligodendrocytes, that are rich in iron-requiring enzymes necessary for myelin production [12,36]—releasing iron into the extracellular matrix subsequently followed by iron uptake into microglia and macrophages [11]. Given its role in (re-) myelination [36], it is subject to discussion that central iron deposition is part of the early repair process in MS, too [12].

A small number of elderly lesions are characterized by prominent T2*w hypointense [5,6,9] as well as phase contrasting rims, too [30]. In contrast to acute lesions with thin phase rims, phase rims of elderly lesions appear to be larger in width [13]. These prominent lesion phase rims are hypothesized to be related to iron-rich microglia phagocytozing intra-lesional debris in a post-inflammatory phase during lesion genesis [13,37].

Nevertheless, lesions in long-standing MS usually showed only marginal phase changes. We hypothesize that intra-lesional phase changes are inversely associated with the age of a lesion which is presumably older in patients with long-standing MS. Theoretical models hypothesized that lesional phase contrast disappears as consequence of neurodegeneration including axonal loss, neurofilament destruction, and integrity loss of the (magnetic) microarchitecture, counteracting a positive phase shift associated with (diamagnetic) myelin loss [14,24,25]. In other words, the diminished visibility of elderly MS lesions on phase images may be a result of positive (paramagnetic) and negative (diamagnetic) phase shifts counteracting each other [24]. Another possible explanation is a lower iron content–and hence a less positive phase shift—in remyelinated lesions and black holes reducing the phase shift caused by demyelination. In consistence with our results, a recent study showed that the susceptibility of older lesions rapidly decreased [37]. A decrease in susceptibility may thus be explained by lower iron concentration rather than remyelination [21].

Additional to phase alterations, our study focused on the detection of small blood vessels centering MS lesions. A central intra-lesional vein was more frequently detectable on T2*w images compared to SWI. Differences presumably derive from a longer echo time applied in our T2*w sequence. Longer echo time increases susceptibility effects caused by the deoxygenated hemoglobin and thus enhances “blooming” of the venous vessel [16,28,38]. Hence, one should focus on adjusting the echo time to T2* of the target region to optimize MR sequences for venous vessel detection.

In order to ensure constant high data quality without influences by spatial resolution constraints and artifacts, this study focused on the morphological description of lesions larger than 5mm diameter. Hence, in some patients only a small number of lesions met inclusion criteria (Fig 3). Nonetheless, an important limitation of this study is its cross-sectional design that does not enable to determine the age of the lesions. Hence, longitudinal studies are warranted to clarify whether SWI-filtered phase imaging can be used to determine the activity and stage of individual MS plaques.

In summary, we observed different patterns of phase changes in MS lesions of patients with short compared to long disease duration. Along with previous studies, we hypothesize that MRI phase shifts during MS lesion evolution. Future comparative studies are warranted to translate our 7T MRI findings to clinical applications (e.g. at 3T MRI).

## Supporting Information

S1 Fig Phase changes in an MS lesion. T2*w MRI (A, B) visualizes a juxtacortical MS lesion (white arrows) not visible on corresponding phase images (C, D).
(TIFF)
