# Supplementary figures and images for: Magnetic Resonance Phase Alterations in Multiple Sclerosis Patients with Short and Long Disease Duration
Source: PLoS One. 2015 Jul 17;10(7):e0128386. doi: 10.1371/journal.pone.0128386 (PMC4506094; doi:10.1371/journal.pone.0128386)

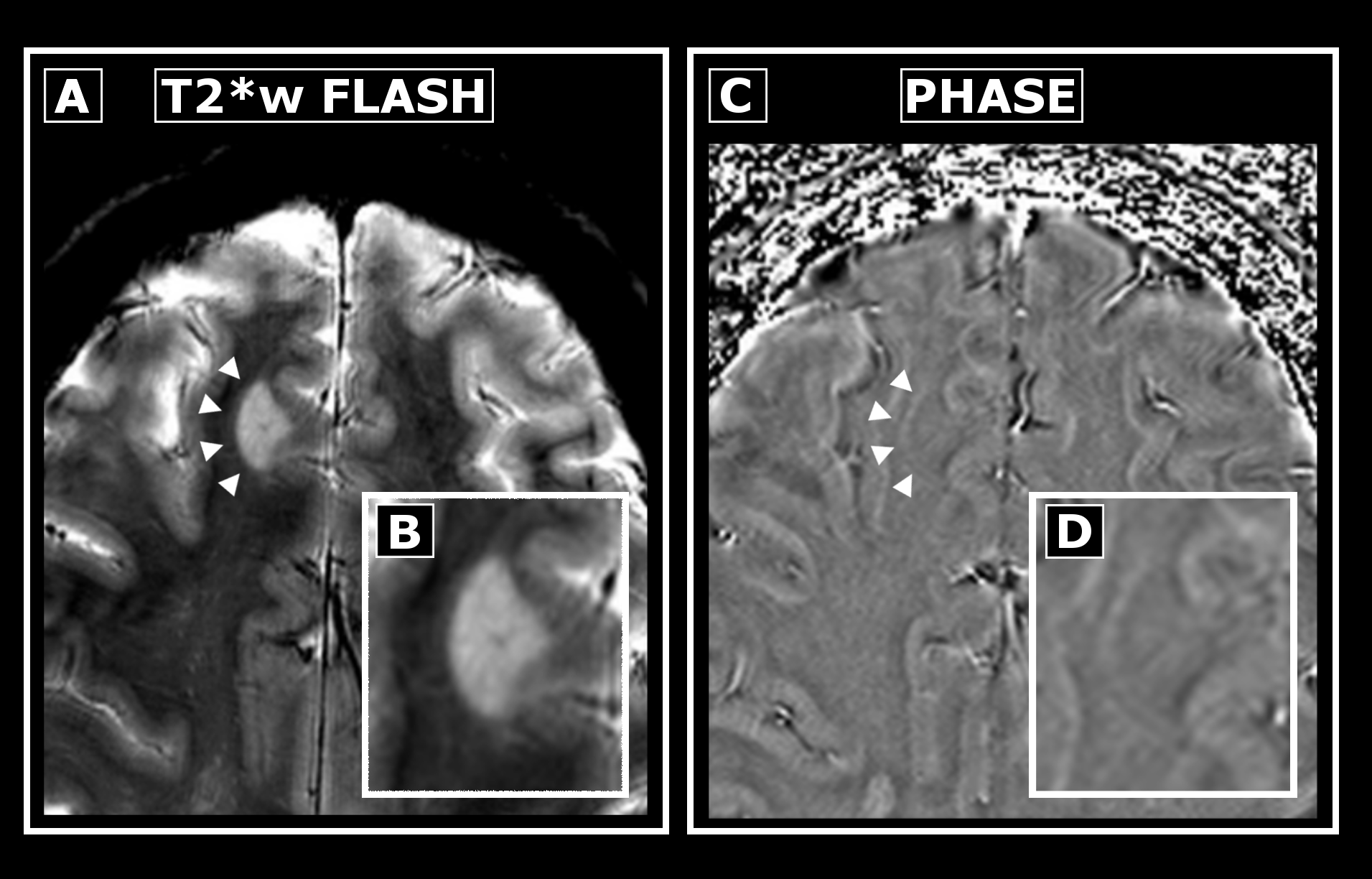

Supplement: S1 Fig — T2*w MRI (A, B) visualizes a juxtacortical MS lesion (white arrows) not visible on corresponding phase images (C, D). (TIFF) [file pone.0128386.s001.tiff]
